# Identification of Alternative Variants and Insertion of the Novel Polymorphic* AluYl17* in* TSEN54* Gene during Primate Evolution

**DOI:** 10.1155/2016/1679574

**Published:** 2016-12-19

**Authors:** Ja-Rang Lee, Young-Hyun Kim, Sang-Je Park, Se-Hee Choe, Hyeon-Mu Cho, Sang-Rae Lee, Sun-Uk Kim, Ji-Su Kim, Bo-Woong Sim, Bong-Seok Song, Kang-Jin Jeong, Youngjeon Lee, Yeung Bae Jin, Philyong Kang, Jae-Won Huh, Kyu-Tae Chang

**Affiliations:** ^1^National Primate Research Center, Korea Research Institute of Bioscience and Biotechnology, Cheongju 28116, Republic of Korea; ^2^National Primate Research Center, Korea Research Institute of Bioscience and Biotechnology, University of Science & Technology (UST), Cheongju 28116, Republic of Korea

## Abstract

*TSEN54* encodes a subunit of the tRNA-splicing endonuclease complex, which catalyzes the identification and cleavage of introns from precursor tRNAs. Previously, we identified an* AluSx*-derived alternative transcript in* TSEN54* of cynomolgus monkey. Reverse transcription-polymerase chain reaction (RT-PCR) amplification and* TSEN54* sequence analysis of primate and human samples identified five novel alternative transcripts, including the* AluSx* exonized transcript. Additionally, we performed comparative expression analysis via RT-qPCR in various cynomolgus, rhesus monkey, and human tissues. RT-qPCR amplification revealed differential expression patterns. Furthermore, genomic PCR amplification and sequencing of primate and human DNA samples revealed that* AluSx* elements were integrated in human and all of the primate samples tested. Intriguingly, in langur genomic DNA, an additional* AluY* element was inserted into* AluSx* of intron eight of* TSEN54*. The new* AluY* element showed polymorphic insertion. Using standardized nomenclature for* Alu* repeats, the polymorphic* AluY* of the langur* TSEN54* was designated as being of the* AluYl17* subfamily. Our results suggest that integration of the* AluSx* element in* TSEN54* contributed to diversity in transcripts and induced lineage- or species-specific evolutionary events such as alternative splicing and polymorphic insertion during primate evolution.

## 1. Introduction

Alternative splicing (AS) can compensate for the lack of an association between gene number and organismal complexity in the mammal genome [1, 2]. By this mechanism, a single gene can produce various transcripts and proteins, contributing to expanding regulatory and functional complexity, protein diversity, and organismal complexity [2]. Previous studies using high-throughput sequencing have reported that >90% of human genes undergo AS in a tissue- or developmental stage-specific manner [3–5]. AS events are classified into several types: exon skipping, alternative 3′ splice site (3′SS), alternative 5′ splice site (5′SS), intron retention, mutually exclusive exons, alternative promoter, and poly(A). Exon skipping, 3′SS, 5′SS, and intron retention events are common types of AS, whereas mutually exclusive exons, alternative promoter, and poly(A) events are less frequent [6–9]. These events can occur when AS sites are recognized or original splicing sites are ignored by the spliceosome [10]. Furthermore, AS events and regulatory mechanisms are highly conserved in mammals [2].

Transposable elements (TEs) are mobile DNA sequences and comprise a large portion of the genome. In humans, TEs comprise 45% of the genome and are contained in introns of about 90% of human genes [11]. TEs provide the AS donor (GT) and acceptor (AG) sites in intron regions, and mature mRNAs contain fragments of TEs through a splicing process called exonization, even within open reading frames (ORFs) [12].* Alu* elements are a common type of TEs in human and nonhuman primate genomes and contribute to new exon creation events [13–16]. The full-length* Alu* element is about 300 nucleotides long, and* Alu* elements are divided into three subfamilies according to the evolutionary time of genome insertion.* AluJ*,* AluS*, and* AluY* are the oldest, intermediate, and youngest subfamilies, respectively.* AluY* elements have transposed most recently, and their novel insertion within a specific genomic locus can generate polymorphisms [17]. Older subfamilies of* Alu *elements commonly lead to exonization. In the human genome,* Alu*-derived new exons or* Alu*-containing exons are found in more than 5% of alternatively spliced exons [13].* Alu*-derived new exons or* Alu*-containing exons allow a protein to establish new functions without affecting the original function [18]. However, most cases of* Alu* exonization occur in UTRs or induce premature transcription termination and do not affect the protein [19]. Nonetheless, the formation of alternative exons from* Alu* can lead to human genetic diseases [20], and they are associated with lineage- or tissue-specific expression during primate evolution [21].

*TSEN54* encodes a subunit of the tRNA-splicing endonuclease complex, involved in the identification and cleavage of introns from precursor tRNAs. This complex is a heterotetramer composed of TSEN2, TSEN34, TSEN15, and TSEN54. An alternatively spliced variant of TSEN2 is part of a complex with unique RNA endonuclease activity [22]. The tRNA-splicing endonuclease complex is also associated with a pre-mRNA 3′ end processing factor [22]. Additionally, depletion of the tRNA-splicing endonuclease complex causes defects in maturation of pre-tRNA and pre-mRNA. Thus, the tRNA-splicing endonuclease complex is involved in multiple RNA-processing events. Previous studies have shown that the* TSEN54 *A307S missense mutation is associated with pontocerebellar hypoplasia (PCH) [23, 24]. Homozygous* TSEN54 *A307S has been identified in PCH type 2 patients, whereas heterozygous* TSEN54 *A307S and a different nonsense mutation are associated with a more severe phenotype consistent with PCH type 4 [25]. However, functional studies of splicing variants or mutants have not yet been performed.

In this study, we focused on the identification and characterization of alternative splicing and exonization events in* TSEN54* in human and nonhuman primates. We performed comparative expression analysis in various cynomolgus, rhesus monkey, and human tissues. Additionally, we analyzed the integration times of* Alu* elements in* TSEN54* during primate evolution.

## 2. Materials and Methods

### 2.1. Ethics Statement

Animal preparation and study design were conducted according to the Guidelines of the Institutional Animal Care and Use Committee (KRIBB-AEC-16067) of the Korea Research Institute of Bioscience and Biotechnology (KRIBB). Rhesus and crab-eating monkeys were provided by the National Primate Research Center of Republic of Korea or imported from China using a Convention on International Trade in Endangered Species of Wild Fauna and Flora permit.

### 2.2. Total RNA and Genomic DNA Samples

Total RNA from pathogen-free, 7-year-old adult, male cynomolgus* (Macaca fascicularis)* monkey tissues (cerebellum, cerebrum, kidney, colon, liver, lung, pancreas, small intestine, spleen, stomach, and testis) and pathogen-free, 10-year-old adult, female rhesus* (Macaca mulatta)* monkey tissues (cerebellum, cerebrum, kidney, colon, liver, lung, pancreas, small intestine, spleen, stomach, and ovary) were extracted using RNeasy® Plus Mini kit (Qiagen). RNA samples from human tissues (bone marrow, whole brain, fetal brain, colon, small intestine, heart, kidney, liver, fetal liver, lung, placenta, prostate, skeletal muscle, spinal cord, spleen, stomach, testis, thymus, trachea, and uterus) were purchased from Clontech Laboratories Inc., USA.

Genomic DNA was isolated using a standard protocol from heparinized blood samples from the following species: (1) Hominoid: chimpanzee* (Pan troglodytes)* and gorilla* (Gorilla gorilla)*; (2) Old world monkey: rhesus monkey* (Macaca mulatta)*, cynomolgus monkey* (Macaca fascicularis)*, African green monkey* (Cercopithecus aethiops)*, colobus* (Procolobus badius)*, and langur* (Trachypithecus cristatus)*; (3) New world monkey: marmoset* (Callithrix jacchus)*, squirrel monkey* (Saimiri sciureus)*, and night monkey* (Aotus trivirgatus)*; (4) Strepsirrhini: ring-tailed lemur* (Lemur catta)*. All primate genomic DNA samples used in this study were provided by the late Professor Osamu Takenaka from the Primate Research Institute of Kyoto University of Japan.

### 2.3. Reverse Transcription-Polymerase Chain Reaction (RT-PCR) and Genomic PCR Amplification

Alternative* TSEN54* transcripts were analyzed by RT-PCR. Moloney Murine Leukemia Virus (M-MLV) reverse transcriptase and an RNase inhibitor (Promega) were used for reverse transcription at a reaction temperature of 42°C. To confirm that the total RNA samples did not contain genomic DNA, we performed PCR using a* TSEN54* primer pair targeted to an intronic region (Figure S1, in Supplementary Material available online at http://dx.doi.org/10.1155/2016/1679574). The RT-PCR reactions consisted of 35 cycles at 94°C for 30 s, 60°C for 30 s, and 72°C for 30 s. Genomic DNA from various primates was used as the template for PCR amplification. Genomic PCR reactions consisted of 30 cycles at 94°C for 30 s, 57°C for 30 s, and 72°C for 30 s. All primers used in this study and their sequences are listed in Table 1.

### 2.4. Molecular Cloning and Sequencing

RT-PCR and PCR products were separated on 1.5% agarose gels, purified with the Gel SV extraction kit (GeneAll) and cloned into the TA cloning vector (RBC Bioscience). Cloned DNA was isolated using a Hybrid-Q™ kit (GeneAll). Sequencing of primate DNA samples and alternative transcripts was performed by Macarogen Inc., Republic of Korea. Nucleotide sequences were aligned using the BioEdit program (http://www.mbio.ncsu.edu/BioEdit/bioedit.html).

### 2.5. Real-Time RT-PCR and Statistical Analyses

*TSEN54* genes, including original transcripts and* Alu*-exonized transcripts, were analyzed by real-time RT-PCR amplification. All real-time RT-PCR primers used and their sequences, are listed in Table 1. Real-time RT-PCR was performed in a Rotor Gene Q thermocycler (Qiagen) for 40 cycles at 94°C for 5 s, 60°C for 10 s. Melting curve analyses were performed for 5 s at 55–99°C. Each sample (1 *μ*L) was added to a 19 *μ*L reaction mixture containing 7 *μ*L H_2_O, 10 *μ*L QuantiTect SYBR Green PCR Master Mix (Qiagen), and 1 *μ*L each of the forward and reverse primers.* TSEN54* amplification efficiencies and correlation coefficients (*R*^2^) were determined from the slopes of the standard curves obtained using a 10-fold serial dilution series. The amplification efficiency was calculated by the following formula: efficiency  (%) = (10^(−1/slope)^ − 1) × 100. Each primer pair exhibited a single, sharp peak indicating that the primers amplified one specific PCR product. Primer dimers were not observed. All target transcripts were normalized for relative quantification by the normalization factor (NF) derived from geometric means delta-Cq (quantification cycles) of the reference genes. All cynomolgus monkey samples were normalized by ADP-ribosylation factor-like 1* (ARL1)*, MORF4 family-associated protein 1* (MRFAP1)*, and ADP-ribosylation factor GTPase activating protein 2* (ARFGAP2)* [26]. All rhesus monkey samples were normalized by ribosomal protein L32* (RPL32)* and ribosomal protein L13a* (RPL13A)* [27]. Hydroxymethylbilane synthase* (HMBS)*, glyceraldehyde-3-phosphate dehydrogenase* (GAPDH)*, and* PRL32* were used as reference genes in human samples [27]. All samples were amplified in triplicate.

## 3. Results and Discussion

### 3.1. Comparative Structure Analysis of the* TSEN54* Gene in Humans and Primates

**Figure 1:**
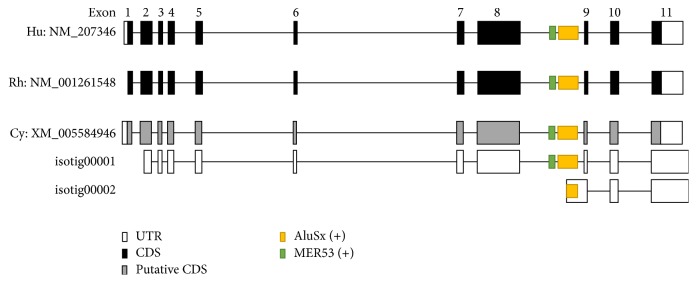
Structural analysis of* TSEN54* of human, rhesus, and cynomolgus monkey. Open, closed black, and gray boxes represent the untranslated region of exons, protein coding region, and putative protein coding region, respectively. The sense directed MER54 and* AluSx* are located on intron 8 of* TSEN54*.

Previously, we analyzed the whole transcriptome of various cynomolgus monkey tissues by RNA sequencing [28] and identified a new AS event, isotig00002, in cynomolgus monkey* TSEN54*. This event occurred by the integration of the* AluSx* element in* TSEN54 *intron 8 (Figure 1). To compare structural differences in* TSEN54* of human, rhesus, and cynomolgus monkey, we analyzed mRNA sequences. Based on the GenBank database, the transcript (NM_207346) of human* TSEN54* gene is composed of 11 exons and is transcribed into a 1970 bp mRNA with a 33 bp 5′ untranslated region (UTR), 1581 bp coding region, and 356 bp 3′ UTR with a consensus polyadenylation signal. The transcript (NM_001261548) of rhesus monkey* TSEN54* is also composed of 11 exons and has 94.1% sequence identity and 96.5% amino acid similarity to human* TSEN54*.* TSEN54* of cynomolgus monkey is still registered as a model RefSeq in the GenBank database.* TSEN54* of human, rhesus, and cynomolgus monkey had structural and sequence homology (Figure 1). Therefore, we focused our investigation on exonization events during primate evolution and comparative expression analysis of original and* Alu*-exonized transcripts.

### 3.2. Validation of the* Alu*-Exonized Transcript and Expression Pattern of the* TSEN54* Gene

**Figure 2:**
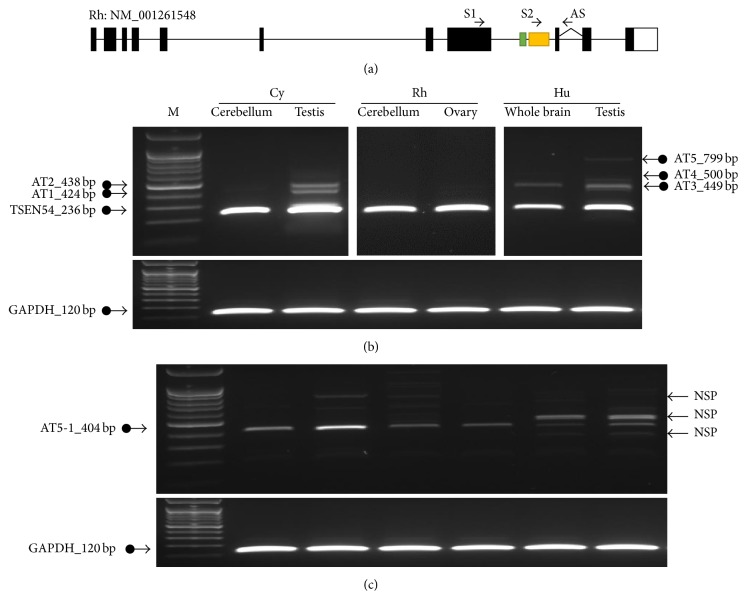
RT-PCR analysis of original and alternative transcripts in cerebellum (or whole brain) and testis (or ovary) tissues of human, rhesus, and cynomolgus monkey. (a) Primer location, RT-PCR amplification using primer 1 pair (b) and primer 2 pair (c).* GAPDH* (120 bp) indicates the positive control. M indicates size marker. AT indicates alternative transcript and NSP is the nonspecific products, confirmed by sequencing.

**Figure 3:**
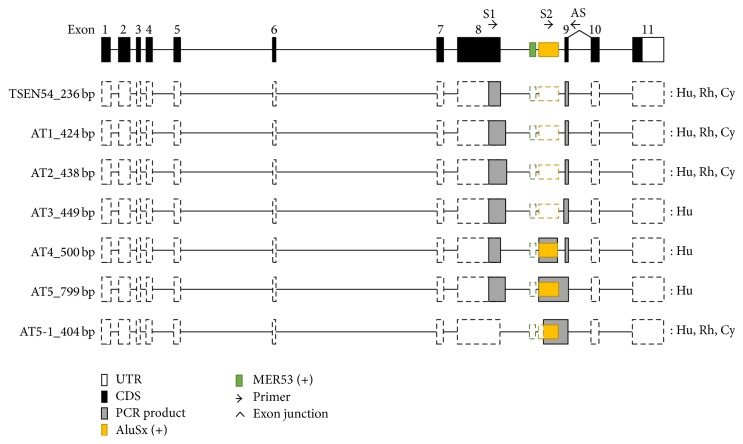
Structure of* TSEN54* original and alternative transcripts. Open, closed black, and gray boxes represent the untranslated region of exons, protein coding region, and PCR product, respectively. Dashed boxes indicate the predicted* TSEN54* gene structure. Arrows indicate primer location.

To identify and validate the* Alu*-exonized transcript, we performed comparative RT-PCR amplification using cerebellum (or whole brain) and testis (or ovary) tissues of human, rhesus, and cynomolgus monkey and sequenced the products. The antisense primer spanned the exon junction between exons 9 and 10, and primer pairs were designed based on the rhesus monkey* TSEN54* gene (Figure 2(a)). Unexpected alternative transcript variants AT1 and AT2 were identified in cynomolgus and rhesus monkey, and AT1–AT5 were identified in human (Figure 2(b)). The sequencing data showed that AT1 and AT2 had an extended form of exon 8 and that AT3 had an extended exon 8 that included about 25 bp of intron 8 (Figure 3). AT4 and AT5 included the* AluSx* sequence as the result of an* Alu*-exonization event. AT5 showed the same structure as isotig00002 identified from cynomolgus monkey RNA seq data. However, AT5 was not detected in cynomolgus or rhesus monkey. Thus, we performed RT-PCR analysis using a primer that included the* AluSx* sequence within* TSEN54* intron 8 (Figure 2(a)). Using this approach, we confirmed the* Alu*-exonized transcript (AT5-1) in cynomolgus and rhesus monkey (Figures 2(c) and 3).

Previous studies have shown that intron-rich or ancient genes are associated with higher AS levels in eukaryotic genomes. Furthermore, ancient gene functions such as RNA-binding and mRNA processing show relatively high levels of AS [29].* TSEN54* functions in mRNA processing and RNA-binding and is an intron-rich ancestral gene. Here, we identified the AS variants of* TSEN54* in human and in rhesus and cynomolgus monkey. Investigation of AS variants between* TSEN54* exons 8 and 9 identified 5 alternative transcripts (Figures 2 and 3). Although identified* TSEN54 *AS variants result in premature termination of transcripts (Figure S2), they were reasonably varied. It was evident that the AS events were activated in* TSEN54*.

The exonization event of many transposable elements, including* Alu,* leads to new exon creation by providing novel splicing sites [12]. Therefore, the alternative splicing machinery contributes to the generation of an abundant transcriptome in primate and human genomes. Recently,* Alu* exonization was shown to be induced by the U2AF65 splicing factor, which is in competition with RNA-binding protein hnRNP C, binding to* Alu* elements [30]. Furthermore, TE-derived exons and transcripts are epigenetically regulated, correlating with cell-type specific gene expression [31]. Exonization of intronic* Alu* elements can induce either cassette exon or exon elongation [32]. In the case of cassette exon, the de novo exon creation occurs by providing both splicing donor and acceptor sites within* Alu* elements. Depending on the location of elongated exons, exon elongation could be subdivided by the simple elongation of an internal exon or the first/last exon.* Alu* exonization could change the original character of a functional gene by providing an alternative promoter, coding sequence, or a premature termination. In this study, the* AluSx* element is exonized in intron 8 of* TSEN54* (Figures 2 and 3). However, the exonization mechanism seems to differ between human and primates. In the human genome,* AluSx* is exonized by two alternative mechanisms of simple elongation and cassette exon (Figure 3). In cynomolgus and rhesus monkey genomes,* AluSx* is exonized only by the simple elongation mechanism. To trace the human-specific cassette exon mechanism, related splice sites were compared using human, cynomolgus, and rhesus monkey genomic sequences (data not shown). The comparison demonstrated that all splice sites are the same across the three genomes. Therefore, the potential of alternative splicing is the same in human, cynomolgus, and rhesus monkey genomes. We concluded that different alternative splicing observed between human and primates was not due to a primate-specific splicing site mutation. The observed splicing differences might be due to splicing regulatory factors, including epigenetic regulation or differences in the binding efficiency of splicing-associated proteins.

**Figure 4:**
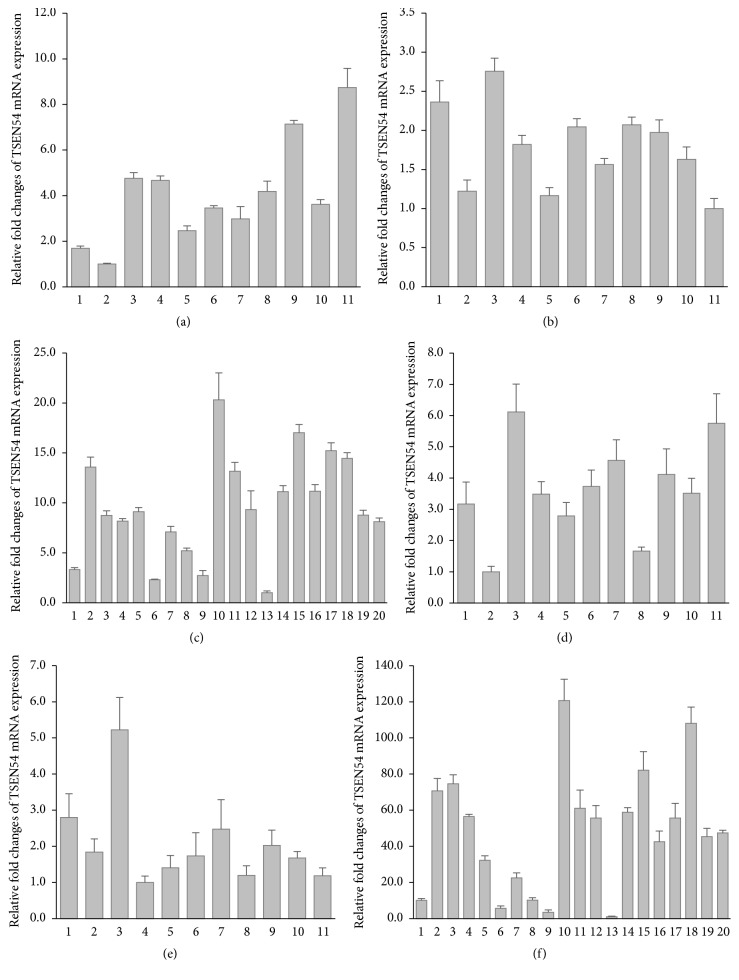
Real-time RT-qPCR analysis of* TSEN54* original and* Alu*-exonized transcripts in various tissues of cynomolgus, rhesus monkey, and human. Expression pattern of* TSEN54* original transcript in cynomolgus monkey (a), rhesus monkey (b), and human (c). Expression pattern of* Alu*-exonized transcript in cynomolgus monkey (d), rhesus monkey (e), and human (f). Cynomolgus monkey panels: 1, cerebellum; 2, cerebrum; 3, kidney; 4, colon; 5, liver; 6, lung; 7, pancreas; 8, small intestine; 9, spleen 10, stomach; 11, testis. Rhesus monkey panels: 1, cerebellum; 2, cerebrum; 3, kidney; 4, colon; 5, liver; 6, lung; 7, pancreas; 8, small intestine; 9, spleen 10, stomach; 11, ovary. Human panels: 1, bone marrow; 2, whole brain; 3, fetal brain; 4, colon; 5, small intestine; 6, heart; 7, kidney; 8, liver; 9, fetal liver; 10, lung; 11, placenta; 12, prostate; 13, skeletal muscle; 14, spinal cord; 15, spleen; 16, stomach; 17, testis; 18, thymus; 19, trachea; 20, uterus.

To understand the expression patterns of the transcript variants of the* TSEN54 *gene including original and* Alu*-exonized transcripts, we performed transcript-specific RT-qPCR in 11 different cynomolgus and rhesus monkey tissues and 20 different human tissues (Figure 4). Original and* Alu*-exonized transcripts of* TSEN54* were ubiquitously expressed in all examined tissues of cynomolgus and rhesus monkey. The original transcript was more highly expressed in human lung tissue, and the* Alu*-exonized transcript was highly expressed in human lung and thymus tissues. Overall, the expression patterns of the original and* Alu*-exonized transcripts were similar. Therefore, we suggested that alternative splicing frequency of original and* Alu*-exonized transcripts was maintained in various tissues. Also,* AluSx* insertion in* TSEN54* gene leads to exonization by creating an alternative splice site but did not affect the expression changes.

### 3.3. Evolutionary Analysis of the* TSEN54* Intron 8* AluSx* Insertion

**Figure 5:**
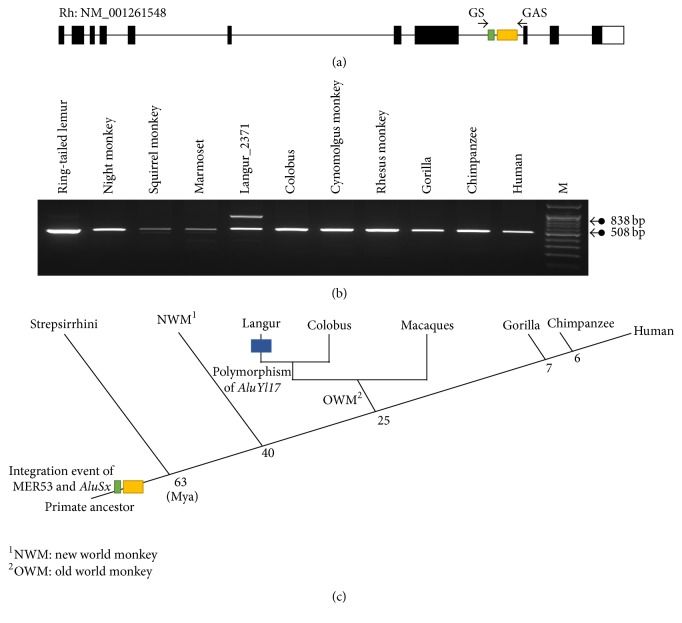
Evolutionary investigation of MER54 and* AluSx* element during primate evolution. (a) Genomic structure of rhesus monkey* TSEN54* gene and primer location. (b) PCR amplification of MER54 and* AluSx* with various primate DNA samples. M indicates the size marker. (c) Schematic representation of the integration events of MER54 and* AluSx* in* TSEN54* during primate evolution. Mya: millions of years ago.

The majority of* Alu* elements were inserted into the genome of the primate common ancestor, 35 to 65 million years ago. During primate evolution, these elements have been amplified to extremely high copy numbers (~500,000 copies) [12]. To measure the integration time of* AluSx* in* TSEN54 *intron 8, we amplified and sequenced this region in the genomic DNA of various primates. PCR amplification targets were* AluSx* and MER53, because MER53 is located within intron 8 of* AluSx* (Figure 5(a)). We identified a 508 bp amplicon containing MER53 and* AluSx* from the* TSEN54* gene in all primate genomic DNA tested, including hominoid (human, chimpanzee, and gorilla), old world monkey (rhesus, cynomolgus monkey, colobus, and langur), new world monkey (marmoset, squirrel, and night monkey), and Strepsirrhini (ring-tailed lemur) (Figure 5(b) and Figure S3). This indicated that MER53 and* AluSx* in* TSEN54* integrated into the genome of a common ancestor before the divergence of Haplorhini and Strepsirrhini (Figure 5(c)), much earlier than 63 million years. Therefore, the* AluSx* element integrated into the primate common ancestor genome and could be a source of alternative splicing during* TSEN54* evolution.

Furthermore, PCR amplification showed an unexpected 838 bp amplicon in langur genomic DNA (Figure 5(b)). Sequencing results indicated that an additional new* AluY* element was inserted into the* AluSx* element of* TSEN54* intron 8 in langur genomic DNA (Figure S3). We assume that the two different PCR amplicons were caused by polymorphic insertion of the* AluY *element in langur. To test our hypothesis, we examined* AluY* element insertion by PCR in two langurs. Two different haplotypes were identified in langur 2371, while two identical haplotypes were identified in langur 2370 (Figure S4). Therefore, we identified the polymorphic insertion of a new* AluY* element in langur genomic DNA (Figure 5(c)).

The* AluY* element is the youngest* Alu* subfamily, and some* AluY* elements have shown polymorphic insertion in the human population [17]. Polymorphic* AluY* have been used as valuable genetic markers for population, linkage, and human identification studies [33, 34]. Previously, human-specific polymorphic* AluYb8* insertion in* WNK1* intron 10 was reported to be associated with blood pressure variation in Europeans [35].* AluYb8* insertion was shown to induce AS events. Here, polymorphic* AluY* insertion in the langur genome occurred inside the exonized* AluSx *of* TSEN54* intron 8, possibly contributing to transcriptomic diversity and complexity via induced AS events. Most* AluY *subfamily elements are associated with direct repeats at flanking regions, called target site duplications (TSDs), of 10–20 bp. TSD sequences can be valuable markers for the confirmation of recent classical target-primed reverse transcription- (TPRT-) mediated* Alu* insertion events [12]. The polymorphic* AluY* element had TSD sequences (GAAAACCTGTCTC) in direct repeats on either side of the element (Figure S4). We suggest that the polymorphic insertion of the AluY element in the langur* TSEN54 *gene is not yet fixed and that this element originated from an active master gene via the TPRT machinery.

### 3.4. Sequence Analysis of Polymorphic* AluY* Element

**Figure 6:**
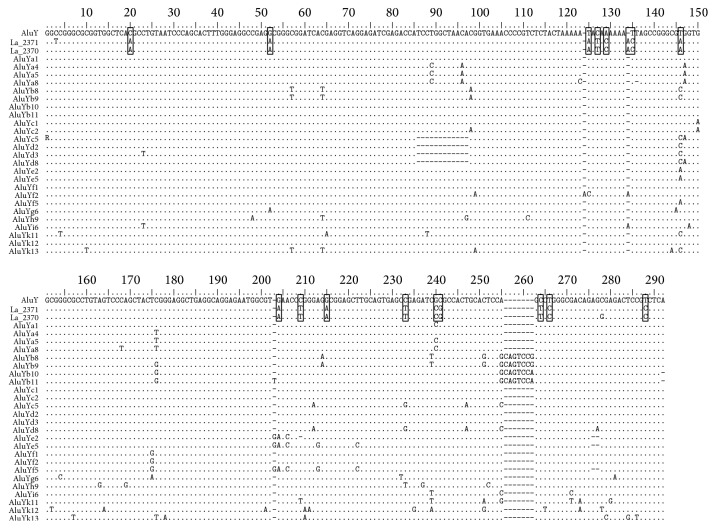
Sequence alignment of various primate* AluY* subfamilies. Various* AluY *subfamilies show the different diagnostic mutation sites.* AluYl17* has seventeen diagnostic mutation sites. Open box is the diagnostic mutation of* AluYl17*. Dots indicate the same sequences with consensus* AluY* sequence and dashes indicate gaps.

Throughout* Alu* evolution in primate genomes, mutations were accumulated within the master genes and subsequently inherited by their copies [36]. These accumulated mutations created new* Alu* subfamilies. Therefore, the* Alu* family is composed of several distinct subfamilies characterized by a hierarchical series of mutations. While the newly amplified* AluY* family is the youngest, it was able to be subdivided and characterized based on diagnostic sites [37]. To classify the subfamily of the polymorphic* AluY* in genomic langur* TSEN54*, we performed sequence analysis. We based our sequence analysis of polymorphic* AluY* and the* AluJ*,* AluS*, and* AluY* subfamilies on Repbase Update (http://www.girinst.org). Polymorphic* AluY* had diagnostic mutation sites common with the consensus* AluY* element and showed 94% sequence identity. However, the polymorphic* AluY* element could not be classified into any existing* AluY* subfamilies. Therefore, we assumed that the polymorphic* AluY* could be part of a new* Alu* subfamily. We found additional 17 specific mutation sites as well as diagnostic mutations of the* AluY* consensus sequences (Figure 6). Therefore, according to standardized nomenclature for* Alu* repeats, the polymorphic* AluY* in langur genomic* TSEN54* could be designated as the* AluYl17* subfamily.

## 4. Conclusions

In this study, we validated and compared exonization derived from* AluSx* on the* TSEN54* gene in human and primate (rhesus and cynomolgus monkey). However, the exonization mechanism seems to differ between human and primates. Next, we confirmed that the* AluSx* element integrated into an intron of the* TSEN54* gene before the divergence of Haplorhini and Strepsirrhini. Furthermore, we identified the polymorphic insertion of a new* AluY* element in the* AluSx* element of langur* TSEN54* gene. Based on our results, we assume that the* AluSx* contributed to diversity in transcripts of* TSEN54* gene by providing an alternative splicing site and induced species-specific evolutionary event such as polymorphic insertion during primate evolution.

## Supplementary Material

Supplementary Figure S1. Verification of no genomic DNA contamination in various cDNA samples. Supplementary Figure S2. Putative translational sequence of alternative transcripts of TSEN54. Supplementary Figure S3. Multiple alignment analysis of human and primate MER54, AluYl17, and AluSx sequences in TSEN54. Supplementary Figure S4. PCR and multiple alignment analysis of polymorphic insertion of AluYl17 in langur TSEN54.

## Figures and Tables

**Table 1:** List of oligonucleotides used in this study.

Name	Sequence	Amplicon size (bp)	Details
RT-PCR for validation of *Alu*-exonized transcript and identification of TSEN54 alternative transcripts			
TSEN54 RT_S1	5′-GGT TCC GGG AAG ATG TCA AC-3′	236	
TSEN54 RT_S2	5′-GCC AAG GTG GGC AGA TCA-3′	404	
TSEN54 RT_AS	5′-CAG CTG GGC ACC TCC ATC-3′		

qRT-PCR for the expression features of *TSEN54* transcripts			
TSEN54 qRT_S1	5′-CAG CTG TGG TCC TTC AGC-3′	198	Targeted to original transcript
TSEN54 qRT_AS1	5′-GGA CAG GCT CAT CAA ATC CAC-3′		
TSEN54 qRT_S2	5′-TCA TGC CAC TAT ACT CCA GCC-3′	213	Targeted to *Alu*-exonized transcript in primate
TSEN54 qRT_AS2	5′-CAG CTG GGC ACC TCC ATC-3′		
TSEN54 qRT_S2-1	5′-GCT AAA TCT GGC CGT CCT AA-3′	112	Targeted to *Alu*-exonized transcript in human
TSEN54 qRT_AS2-1	5′-AGC ACA GAG ATA TGC TGA AGG A-3′		

qRT-PCR reference genes			
ARL1 qRT_S	5′-AGA CAG TTG TGA CCG AGA CC-3′	136	Cynomolgus monkey
ARL1 qRT_AS	5′-TGA GGA AGT CAT GGC CTG TT-3′		
MRFAP1 qRT_S	5′-GCG GAT AGA GAA GAG CGA GT-3′	82	
MRFAP1 qRT_AS	5′-AGC CAA TCT CCA CCA GTT GA-3′		
ARFGAP2 qRT_S	5′-GCG TCC ATC TGA GCT TCA TC-3′	135	
ARFGAP2 qRT_AS	5′-CAT CAT TGG CTG TGC ATC CA-3′		
RPL32 qRT_S	5′-CAA CAT TGG TTA TGG AAG CAA CA-3′	80	Rhesus monkey and human
RPL32 qRT_AS	5′-TGA CGT TGT GGA CCA GGA ACT-3′		
RPL13A qRT_S	5′-CCT GGA GGA GAA GAG GAA AGA GA-3′	126	Rhesus monkey
RPL13A qRT_AS	5′-TTG AGG ACC TCT GTG TAT TTG TCA A-3′		
HMBS qRT_S	5′-ACC AAG GAG CTT GAA CAT GC-3′	145	Human
HMBS qRT_AS	5′-GAA AGA CAA CAG CAT CAT GAG-3′		
GAPDH qRT_S	5′-GAA ATC CCA TCA CCA TCT TCC AGG-3′	120	
GAPDH qRT_AS	5′-GAG CCC CAG CCT TCT CCA TG-3′		

Genomic DNA PCR for evolutionary analysis of *AluSx *insertion			
TSEN54 GS	5′-ATG GGA ATG CGG TAG ATT GT-3′	508	
TSEN54 GAS	5′-AGG GGA GTC ACA TTT CTC AGT C-3′		
