# Supplementary figures and images for: Identification of Alternative Variants and Insertion of the Novel Polymorphic AluYl17 in TSEN54 Gene during Primate Evolution
Source: Int J Genomics. 2016 Dec 19;2016:1679574. doi: 10.1155/2016/1679574 (PMC5204098; doi:10.1155/2016/1679574)

## Slide 1
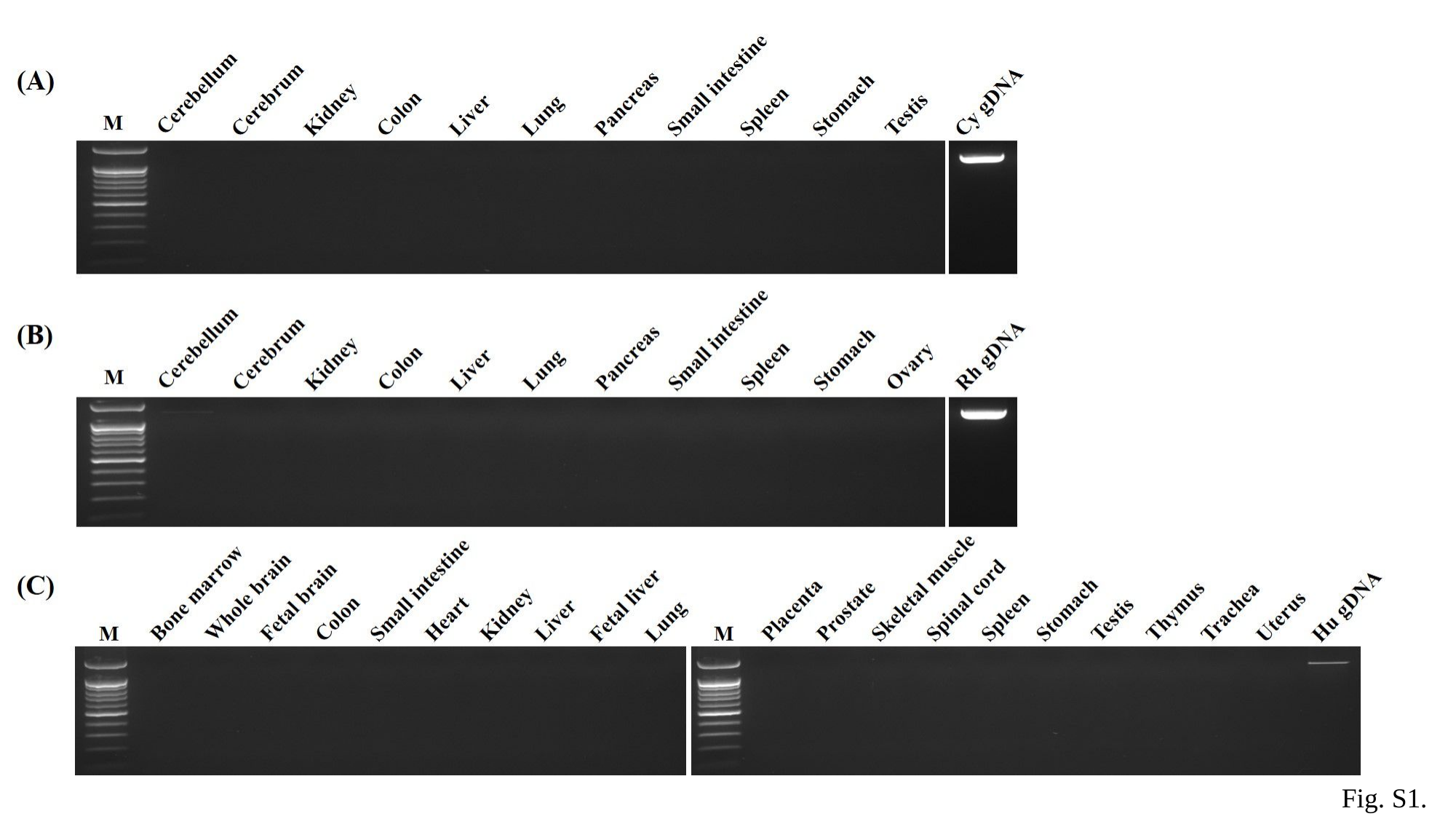

Fig. S1.

## Slide 2
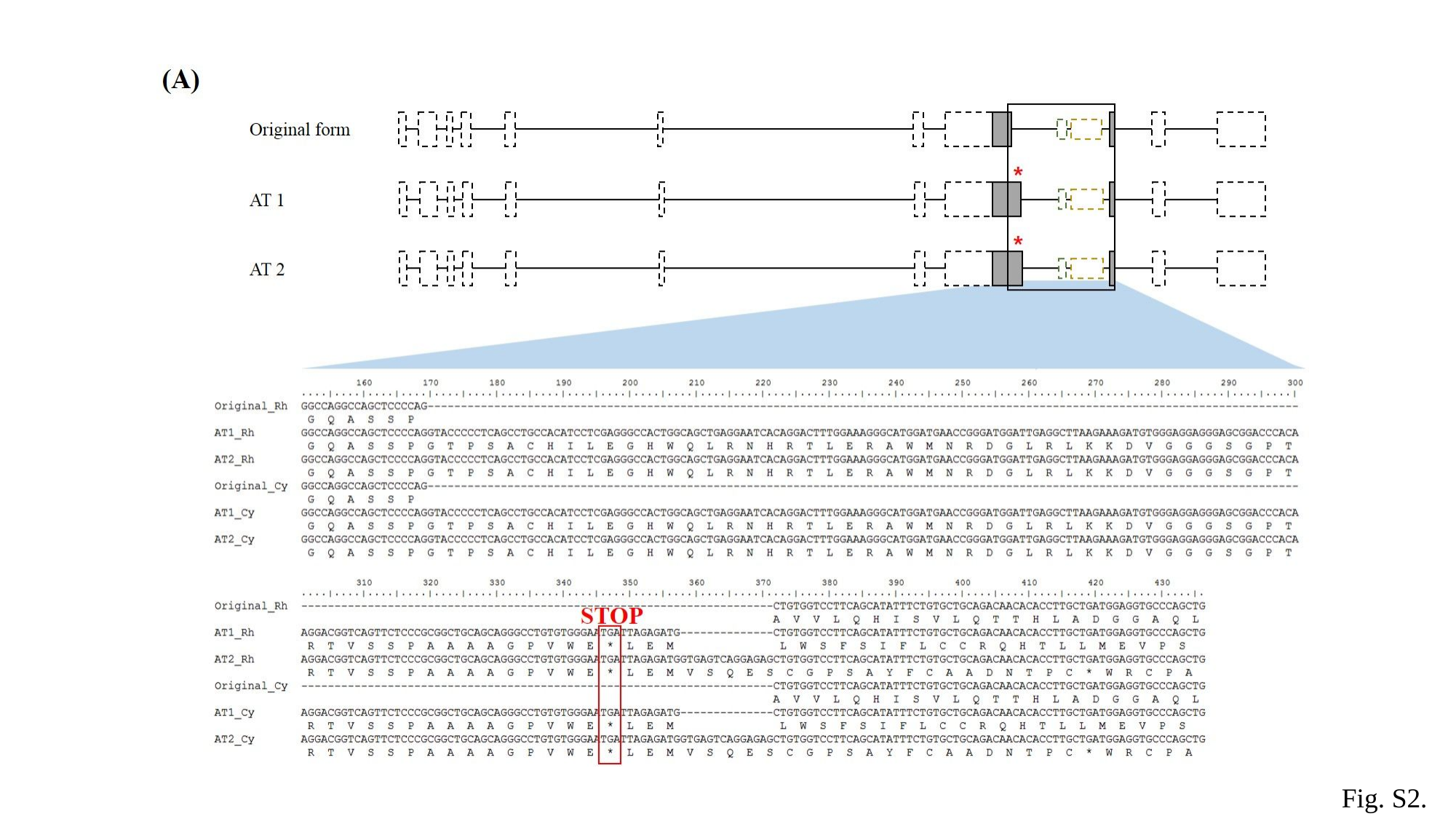

Fig. S2.

## Slide 3
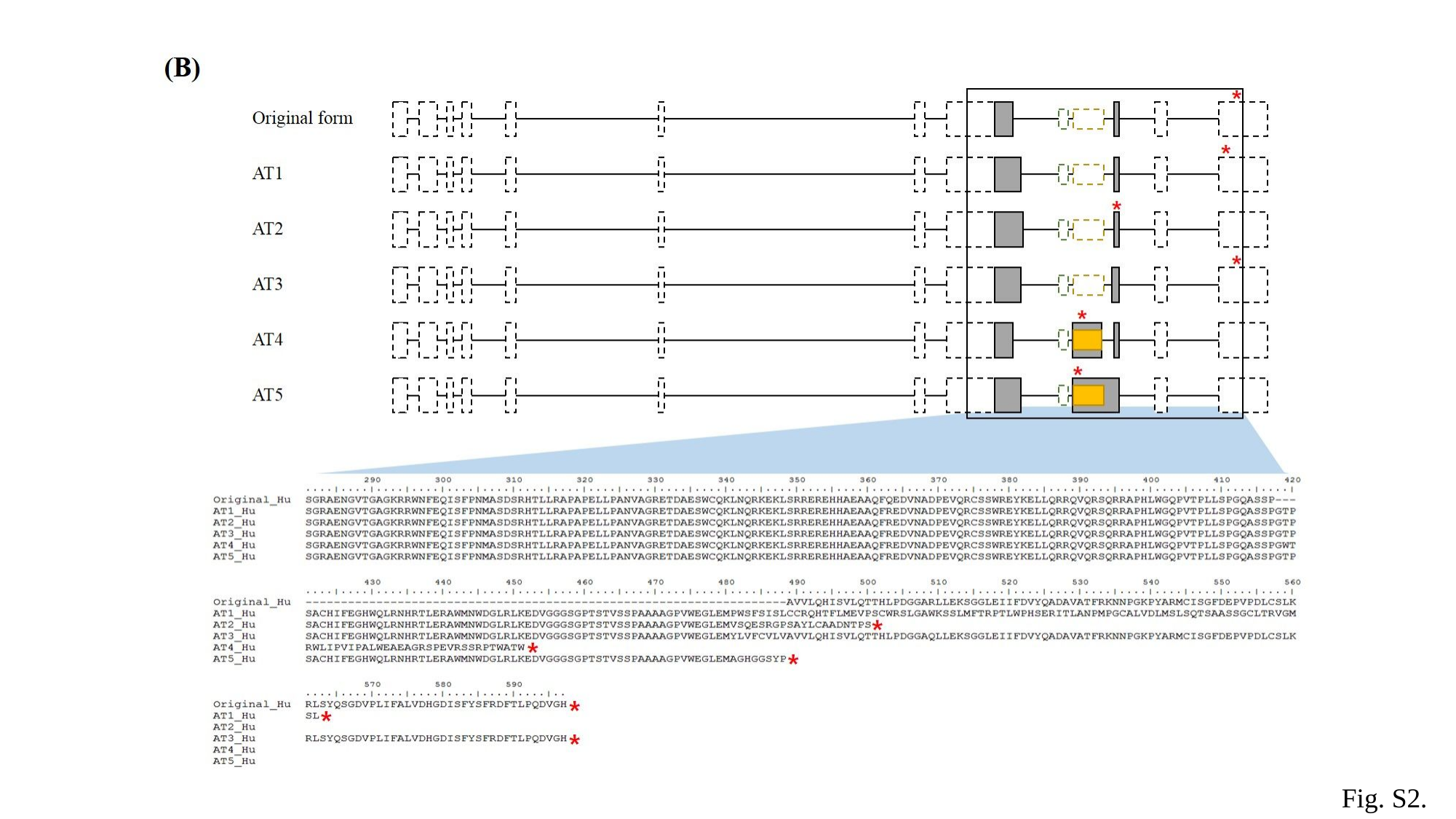

Fig. S2.

## Slide 4
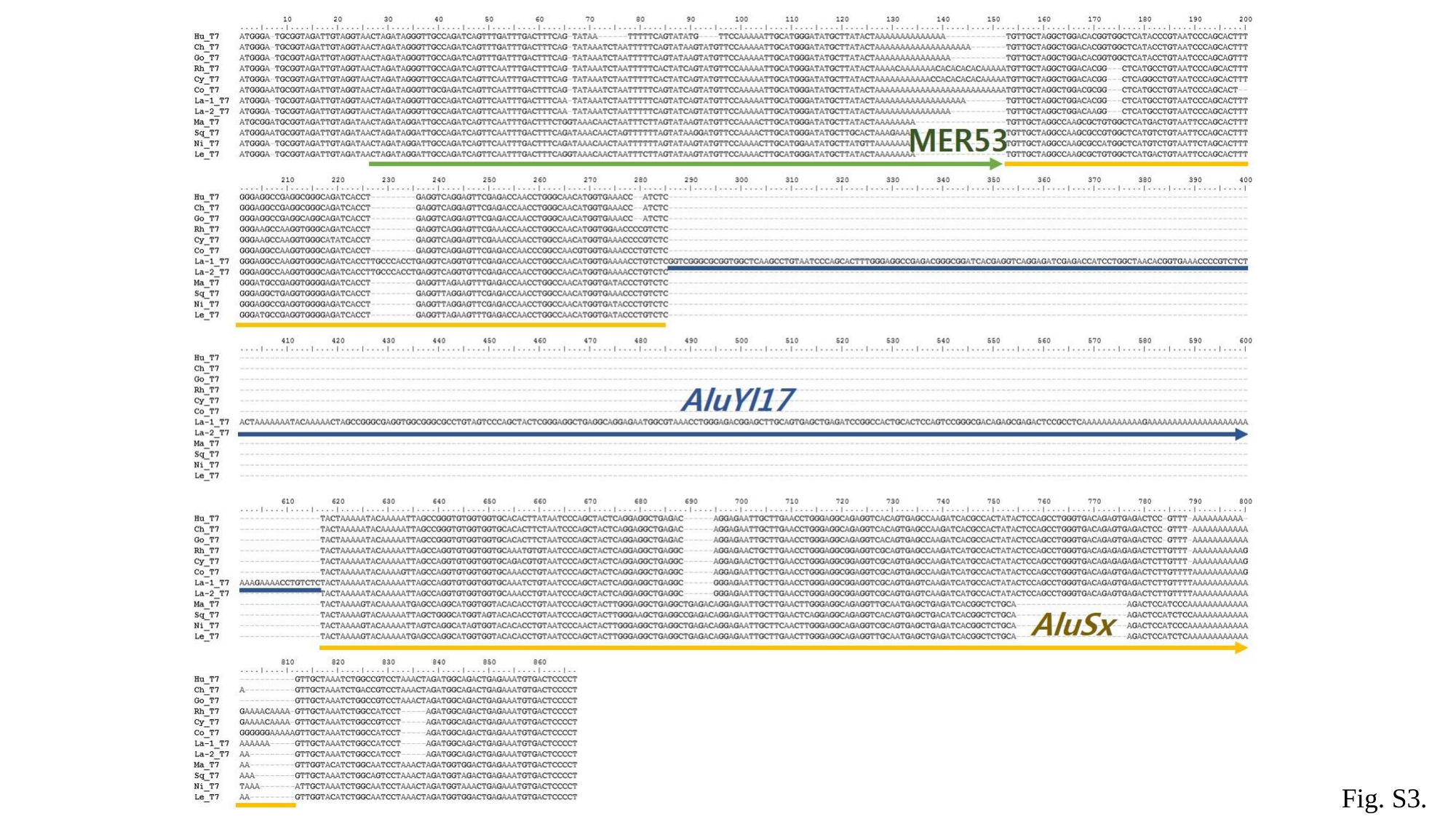

Fig. S3.

## Slide 5
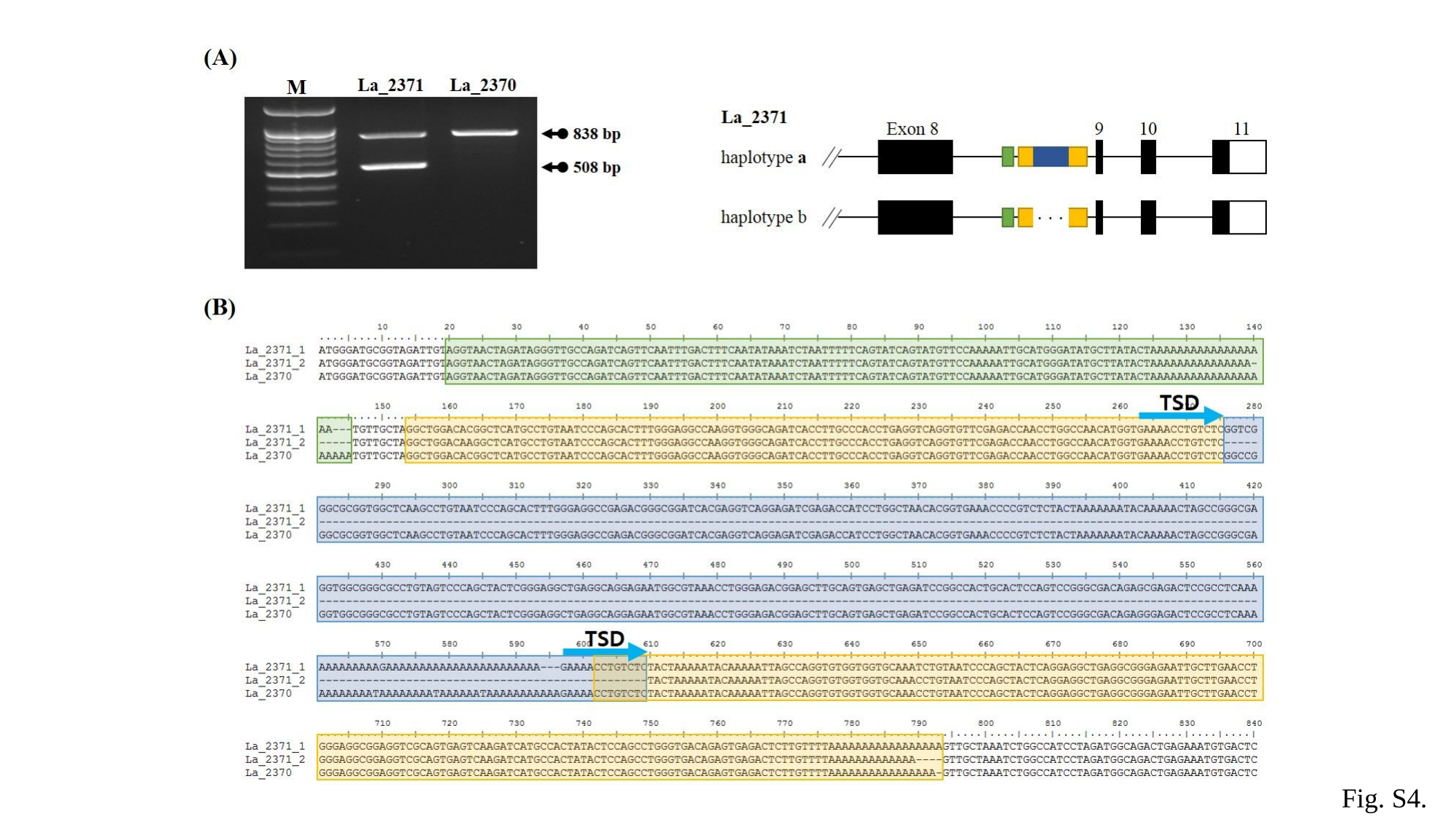

Fig. S4.

Supplement: Supplementary file 1 — Supplementary Figure S1. Verification of no genomic DNA contamination in various cDNA samples. Supplementary Figure S2. Putative translational sequence of alternative transcripts of TSEN54. Supplementary Figure S3. Multiple alignment analysis of human and primate MER54, AluYl17, and AluSx sequences in TSEN54. Supplementary Figure S4. PCR and multiple alignment analysis of polymorphic insertion of AluYl17 in langur TSEN54. [file 1679574.f1.pptx]
